# Hereditary Hemorrhagic Telangiectasia: Diagnosis and Management

**DOI:** 10.3390/jcm11164698

**Published:** 2022-08-11

**Authors:** Cuesta M. Angel

**Affiliations:** 1 Departamento de Bioquímica y Biología Molecular, Facultad de Farmacia, Complutense University of Madrid, 28040 Madrid, Spain; angcuest@ucm.es; 2 CIBERER, Centro de Investigación Biomédica en Red de Enfermedades Raras, Unidad 707, Instituto de Salud Carlos III (ISCIII), 28029 Madrid, Spain

Hereditary hemorrhagic telangiectasia (HHT), or Rendu-Osler-Weber syndrome, is a dominantly inheritable rare disease with a prevalence of 1:5000–10,000 inhabitants.

The diagnosis of HHT follows the Curaçao diagnostic criteria [1]:

1. NOSEBLEEDS (epistaxis) that are spontaneous and recurrent.
2. (Multiple) TELANGIECTASES at characteristic sites, including the lips, oral cavity, fingers and nose.
3. INTERNAL LESIONS: arteriovenous malformations (AVMs) or telangiectases in the stomach, lungs, liver, brain and spinal cord.
4. FAMILY HISTORY: a first-degree relative with HHT according to these criteria.

When the patient meets at least three of these criteria, he/she is considered to have definitive HHT.

To date, three subtypes of HHT have been described. HHT type 1 refers to mutations of the endoglin gene *ENG*; HHT type 2 refers to mutations of the activin A receptor, similar to the type-1 *ACVRL1* gene; and the third type, known as juvenile polyposis–hereditary hemorrhagic telangiectasia (JPHT or JPHHT) overlap syndrome, refers to mutations of the gene *MADH 4*. There are two other subtypes (HHT-3 and HHT-4) whose mutations have not yet been completely identified, but it is known that they are located in the 5q31.3–q32 and 7p14 chromosomal regions, respectively [2,3].

This Special Issue (SI), with nine original articles and one review, focuses on “Diagnosis and Management.” Management is not possible without a correct diagnosis. However, this obvious statement ignores the fact that the average time taken for a diagnosis of HHT to be established is 27 years, as noted by Major, T. et al., with the average diagnosis in Hungary being obtained over periods between 22.6 and 29.1 years [4].

This phenomenon occurs in developed countries, but we cannot forget that we still need to address the terra incognita. We cannot forget about developing or emerging countries, where the prevalence should be similar, and the founder effects may be reinforced by physical boundaries [5], but the medical assistance is far from the desired level.

In this sense, the international collaboration reported by Errasti Díaz et al. highlights the difficulties of performing a rather easy genetic test for a new mutation HHT in Peru [6]. In addition to the novelty of this new mutation, it should also shed light on and inspire international collaboration, due to the fact that the number of inhabitants can lead to thousands of HHT patients (and patients with many other rare diseases) remaining unidentified.

Major, T. et al. assert that society is lacking in knowledge of HHT disease and that this is related to the unawareness of HHT within the medical community [4]. I would only add that, as seen in this SI, those physicians who are aware of rare diseases such as HHT they never let their patients down and always do their best to help them.

Gaetani E. et al. describe a very interesting genotype–phenotype correlation between cerebrovascular malformations in HHT patients. This kind of study could help to eliminate the differences identified in 2020 regarding the recommendations for screening children for brain VMs [7,8].

Andorfer et al. present a retrospective study on the evolution and management of HHT disease in pregnant HHT patients, indicating that, even that the risks are low, better procedures should be implemented to minimize the negative effects of the disorder [9].

COVID-19, as is the case worldwide, has also affected HHT patients. In this SI, one can read about an interesting Spanish study, in which HHT patients appeared to be less affected by the viral infection due to a minor cytokine storm [10]. It would be incredibly useful if a larger and international retrospective study were to confirm these data, which were obtained from 138 HHT patients. Moreover, the results of an Italian observational study on the remote care of HHT patients identified the benefits and rapid response of this kind of assistance [11].

Lastly, but not least, this SI reports on different aspects of the management of HHT patients aiming to improve their quality of life. In Germany, Seebauer et al. studied the benefits of using an HHT disease calendar, indicating the improvement of the cross-communication between patients and clinicians, but also highlighting the lack of knowledge about the disease among the patients, as indicated previously in [12]. Droege et al. described the underestimation of restless leg syndrome in HHT patients as a consequence of recurrent epistaxis and internal bleedings and highlighted the need for a treatment [13].

Moreover, the management of this disease for a better quality of life of the patients involves the improvement or the creation of simple, rapid and cheap treatments. In this sense, Marcos et al. demonstrated the benefits of sclerotherapy on demand, as observed in 105 patients [14].

Finally, we can conclude that this SI covers key areas of the disease that must be solved in the near future, such as the need for international collaboration, the management of cerebrovascular malformations, and the improvement of the daily life of the patients through educational programs on the disease, remote care and specialized on-demand assistance.

We hope that our readers enjoy this SI, which was revised by international experts in the basic research and clinics of HHT disease.
